# The effects of dipeptidyl peptidase-4 inhibitors on cardiac structure and function using cardiac magnetic resonance: a meta-analysis of clinical studies

**DOI:** 10.3389/fendo.2024.1428160

**Published:** 2024-09-11

**Authors:** Haipeng Wang, Siyi Guo, Shuo Gu, Chunyu Li, Fei Wang, Junyu Zhao

**Affiliations:** ^1^ Shandong Provincial Hospital, Shandong University, Jinan, Shandong, China; ^2^ The First Clinical Medical College, Cheeloo College of Medicine, Shandong University, Jinan, Shandong, China; ^3^ Department of Endocrinology and Metabology, Shandong Provincial Qianfoshan Hospital, Jining Medical University, Shandong Key Laboratory of Rheumatic Disease and Translational Medicine, Shandong Institute of Nephrology, Jinan, Shandong, China; ^4^ Department of Endocrinology and Metabology, The First Affiliated Hospital of Shandong First Medical University and Shandong Provincial Qianfoshan Hospital, Shandong Key Laboratory of Rheumatic Disease and Translational Medicine, Shandong Institute of Nephrology, Jinan, Shandong, China; ^5^ Department of Endocrinology and Metabology, Shandong Provincial Qianfoshan Hospital, Cheeloo College of Medicine, Shandong University, Jinan, Shandong, China

**Keywords:** dipeptidyl peptidase-4 inhibitors(DPP4i), type 2 diabetes mellitus(T2DM), cardiac magnetic resonance(CMR), cardiac structure and function, meta-analysis

## Abstract

**Objective:**

The aim of the study was to evaluate the effect of dipeptidyl peptidase-4 inhibitors (DPP4i) on cardiac structure and function by cardiac magnetic resonance (CMR). Research Methods & Procedures: Database including PubMed, Cochrane library, Embase and SinoMed for clinical studies of DPP4i on cardiac structure and function by CMR were searched. Two authors extracted the data and evaluated study quality independently. Mean difference (MD) or standardized MD and 95% confidence intervals (CI) were used for continuous variables. Review Manager 5.3 was used to performed the analysis.

**Results:**

Ten references (nine studies) were included in this meta-analysis. Most of the studies were assessed as well quality by the assessment of methodological quality. For clinical control studies, the merged MD values of △LVEF by fixed-effect model and the pooled effect size in favor of DPP4i was 1.55 (95% CI 0.35 to 2.74, P=0.01). Compared with positive control drugs, DPP4i can significantly improve the LVEF (MD=4.69, 95%CI=2.70 to 6.69), but no such change compared to placebo (MD=-0.20, 95%CI=-1.69 to 1.29). For single-arm studies and partial clinical control studies that reported LVEF values before and after DPP4i treatment, random-effect model was used to combine effect size due to a large heterogeneity (Chi^2^ = 11.26, P=0.02, I^2^ = 64%), and the pooled effect size in favor of DPP4i was 2.31 (95% CI 0.01 to 4.62, P=0.05). DPP4i significantly increased the Peak filling rate (PFR) without heterogeneity when the effect sizes of two single-arm studies were combined (MD=31.98, 95% CI 13.69 to 50.27, P=0.0006; heterogeneity test: Chi^2^ = 0.56, P=0.46, I^2^ = 0%).

**Conclusions:**

In summary, a possible benefit of DPP4i in cardiac function (as measured by CMR) was found, both including ventricular systolic function and diastolic function.

## 1 Introduction

Patients with type 2 diabetes mellitus (T2DM) have a more than doubled risk of developing cardiovascular disease (CVD) than those without (1). More than half of the deaths in DM patients are caused by CVD (2). In addition to CVD, the pathogenesis of cardiac dysfunction caused by diabetes is quite complex, involving cellular, molecular and structural abnormalities. Diabetic cardiomyopathy is also a very important mechanism and is a type of systolic and diastolic dysfunction different from diabetic microangiopathy. Early active and effective hypoglycemic therapy can reduce the risk of complications (including microvascular and macrovascular events) or death in newly diagnosed diabetic patients (3–7). Since 2008, none of the hypoglycemic drugs have shown any cardiovascular safety concerns compared to placebo. Even some hypoglycemic drugs have shown unique cardiovascular benefits (8). Numerous clinical studies have shown evidence of cardiovascular benefits of glucagon-like peptide 1 (GLP-1) receptor agonist compared to placebo (9–15). Dipeptidyl peptidase-4 inhibitors (DPP4i), one of the commonly used hypoglycemic drugs, can improve blood glucose control in T2DM patients by inhibiting the degradation of glucopeptide-1 and glucopeptide-dependent insulin polypeptides, prolongating the action time of endogenous hormones, inhibiting glucagon levels and increasing endogenous insulin secretion (16). However, the cardiovascular effects of DPP4i in patients with diabetes remain unclear. Some studies have shown that DPP4i reduce the risk of adverse cardiovascular events, while others have been neutral about this effect (17–19).

For clinical trials, endocardial biopsy is the main method to evaluate the changes of myocardial pathological in patients. However, due to such shortcomings as the invasiveness, sampling errors, serious complications and poor consistency between observers, evidence of myocardial pathological in heart disease in human is rare (20, 21). For patients with heart diseases or high risk of heart diseases, it is important to evaluate myocardial tissue or cardiocytes by a non-invasive technique. In recent years, cardiac magnetic resonance (CMR) has been widely used in the diagnosis and prognosis assessment of various heart diseases, and is the most commonly used imaging method to evaluate myocardial damage such as myocardial edema and fibrosis (22, 23). Previous studies have found myocarditis (24, 25), dilated cardiomyopathy (26), myocardial infarction (27, 28), heart failure (29, 30) and other heart diseases that have potential myocardial damage by CMR technology, and they were well correlated with myocardial histopathological changes. Early monitoring of global or local myocardial systolic function abnormalities provides important information for early diagnosis and prognosis evaluation of cardiovascular diseases and cardiomyopathy (31). Appling CMR, a sensitive and non-invasive technique, to evaluate the benefit of hypoglycemic drugs, especially the controversial DPP4i, in heart disease may be a good suggestion.

Therefore, this study searched and summarized the previous application of CMR technology to verify the effects of DPP4i on cardiac structure and function, so as to bring better evidence-based medical evidence for the cardiac benefits of DPP4i.

## 2 Materials and methods

This meta-analysis was conducted under the guidance of the Preferred Reporting Items Statement for Systematic Evaluation and Meta-Analysis (PRISMA).

### 2.1 Searching progress

We searched the following databases for clinical studies of DPP4i on cardiac structure and function by CMR: PubMed, Cochrane library, Embase and SinoMed, for clinical studies. A list of references to all eligible articles and related review articles was also manually searched. A literature search of this meta-analysis was limited to published results. Databases were searched from the earliest data to 29 January 2024 with the following search terms: (“Dipeptidyl-Peptidase IV Inhibitors” OR “DPP-4 Inhibitor*” OR “DPP 4 inhibitor*” OR “DPP4 inhibitor*” OR “Dipeptidyl peptidase-4 inhibitor*” OR “DPP-IV Inhibitor*” OR “DPP IV Inhibitor*” OR “saxagliptin” OR “sitagliptin” OR “vildagliptin “ OR “linagliptin” OR “alogliptin” OR “anagliptin” OR “gemigliptin” OR “teneligliptin”) AND (“Cardiac Imaging Techniques” OR “CMR” OR “cardiac magnetic resonance” OR “cardiac MRI imaging” OR “cardiac MR imaging”). Eligible studies were screened and selected based on the following criteria (1): published in English or Chinese (2); evaluated the effect of DPP4i on cardiac structure and function by CMR (3); clinical study (either clinical control study or single-arm study) (4); reported at least one outcome of cardiac structure and function by CMR.

### 2.2 Study selection and data extraction

The studies were screened independently by two authors, and any differences were resolved by consensus. If there is still doubt, a third experienced author was invited to join the consultation and reach a consensus finally. The following data were extracted from the eligible studies (1): characteristic of populations, interventions, number of participants (2); follow-up time (3); MR system (4); outcome index.

### 2.3 Methodological quality assessment

Risk of bias is used for randomized/clinical control studies to assess the methodological quality. A designed tool for assessing risk of bias in single-arm studies considered the following items: selection bias, lead time bias/immortal time bias, confounding by indication, misclassification bias/information bias, bias from natural recovery/regression to the mean, bias due to adjunctive therapies, attrition bias, selective reporting of outcomes. For risk of bias table, a point of 4 or less is considered “poor methodological quality”, while a point above 4 is defined as “good methodological quality”. Two authors scored these items independently.

### 2.4 Statistical analysis

The main outcome was the change of LVEF over the treatment duration. We also analyzed the change of other index that reflect the cardiac structure and function (including left ventricular function parameters, right ventricular function parameters, heart fat content and myocardial strain parameter) by CMR technique. For continuous variables, mean difference (MD), standardized MD and 95% confidence intervals (CI) were used. Fixed-effect model was used for data analysis. The I^2^ was calculated as an indicator of the inter-study heterogeneity. If the heterogeneity test showed a large heterogeneity, the random-effects model was replaced. All data analysis was performed by Review Manager 5.3 (Cochrane Collaboration, United Kingdom, http://www.cochrane.org).

## 3 Results

### 3.1 Search results and characteristics of included studies

**Figure 1:**
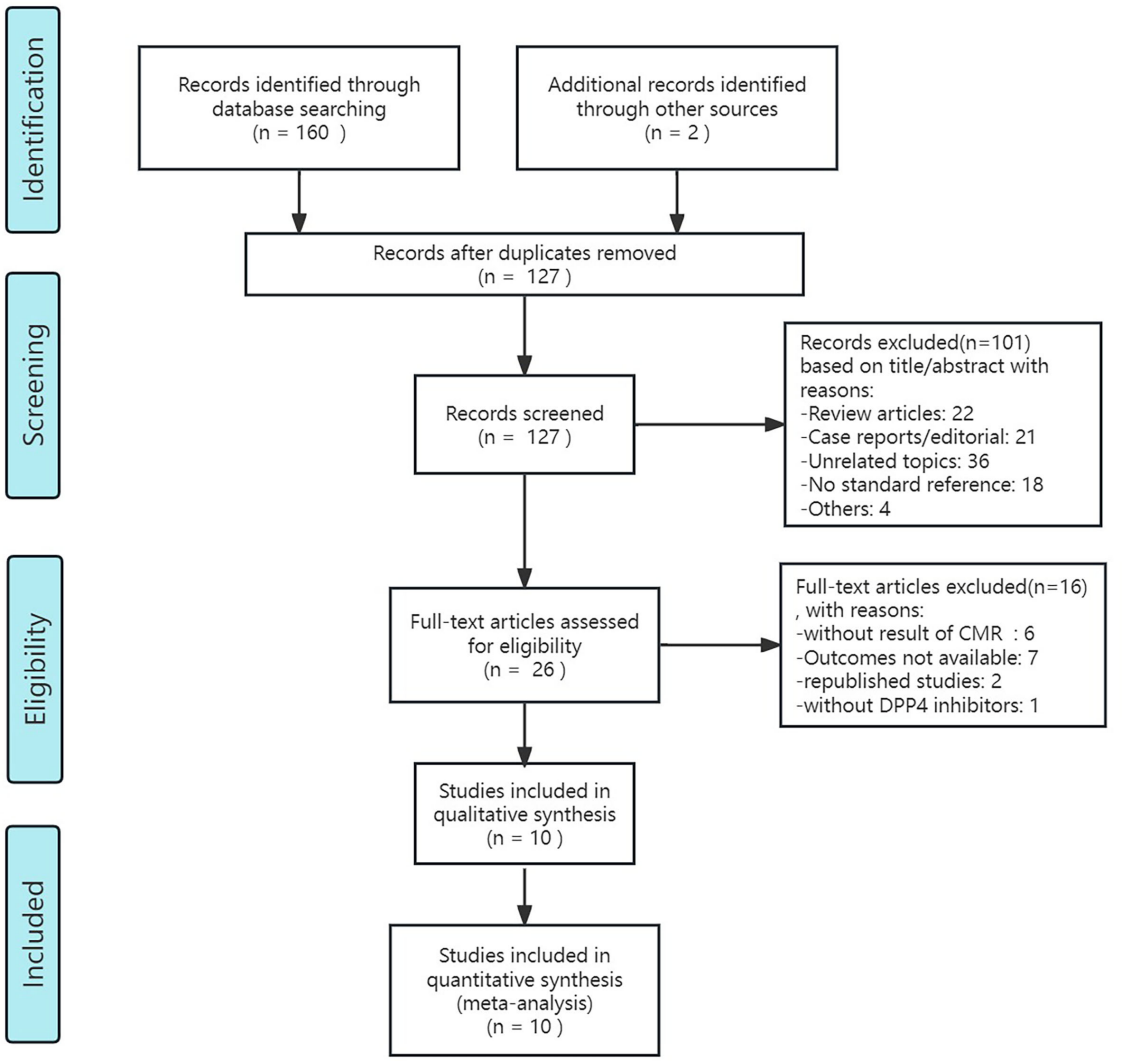
Flow chart of the systematic search process.

After retrieval form the database showed above and re-checking, 127 articles of potentially relevant studies need further classified. After screening the abstract, 26 articles were required to be read in full. Of these, 10 articles (9 studies) were eligible (32–41). **Figure 1** shows the searching progress. Seven studies are published in English (34–41), the rest two were Chinese (32, 33). Two countries, China and Japan, each had two studies included. Canada, the UK, Germany and Australia each had one study included. And a clinical study conducted simultaneously in several countries around the world was also included in the article. Two of the studies included patients with myocardial infarction (36, 41), while the remaining studies included patients with type 2 diabetes. These studies included diabetic patients with heart failure (37, 40) or without history of heart failure (32–34, 39), or with known or suspected cardiovascular disease (38), or obese T2DM patients (35). In the nine included studies, different DPP4i were applied, including saxagliptin, sitagliptin, alogliptin and dutogliptin, and the doses vary. Totally, for clinical control studies, there are 347 patients received DPP4i treatment and 346 patients assigned to the control group. And the sample size ranges from 10 to 112 in DPP4i treatment group while 10 to 120 in control group (32, 35, 36, 38–41). For single-arm studies, 32 patients were included totally (33, 34, 37). The follow-up ranged from 28 days to 26 weeks. **Table 1** summarizes the detailed characteristics of these nine included studies.

**Table 1:** Characteristic of nine included studies.

First author, year	Country	Populations	Comparations		Sample size		Follow-up
			Experimental group	Control group	Experimental group	Control group	
NCT02917031	Globally	T2DM patients with heart failure	saxagliptin	placebo	112	120	24 weeks
Christoph Brenner, 2016	Germany	NSTEMI patients	sitagliptin 100mg/day	placebo	87	86	28 days
Shingo Kato, 2016	Japan	T2DM patients with known or suspected CAD	alogliptin	glimepiride	10	10	3 months
Xiaofang Wang, 2018	China	Newly diagnosed T2DM patients with normal systolic function	sitagliptin	non-DPP4i	58	59	12 months
David R. Webb MBChB, 2020	UK	Obese T2DM patients	sitagliptin 100mg qd	liraglutide	33	28	26 weeks
Shigenori Hiruma, 2021	Japan	T2DM patients	sitagliptin 50-100mg/day	empagliflozin 50mg/day	21	21	12 weeks
Paul Sandhu, 2021	Canada	T2DM patients without a preexisting history of heart failure	saxagliptin 5mg qd	/	16	/	6 months
Kathy CK. Wong, 2024							
Likong Fu, 2022	China	T2DM patients without a preexisting history of heart failure	saxagliptin 5mg qd	/	16	/	6 months
Dirk von Lewinski, 2022	Austria	Successfully treated STEMI but reduced LVEF	dutogliptin 60mg bid	placebo	26	22	90 days

T2DM, type 2 diabetes mellitus; NSTEMI, non-ST-elevation myocardial infarction; CAD, coronary artery disease; STEMI, ST-elevation myocardial infarction; LVEF, left ventricular ejection fraction; MR, magnetic resonance.

### 3.2 Quality assessment of included studies

**Figure 2:**
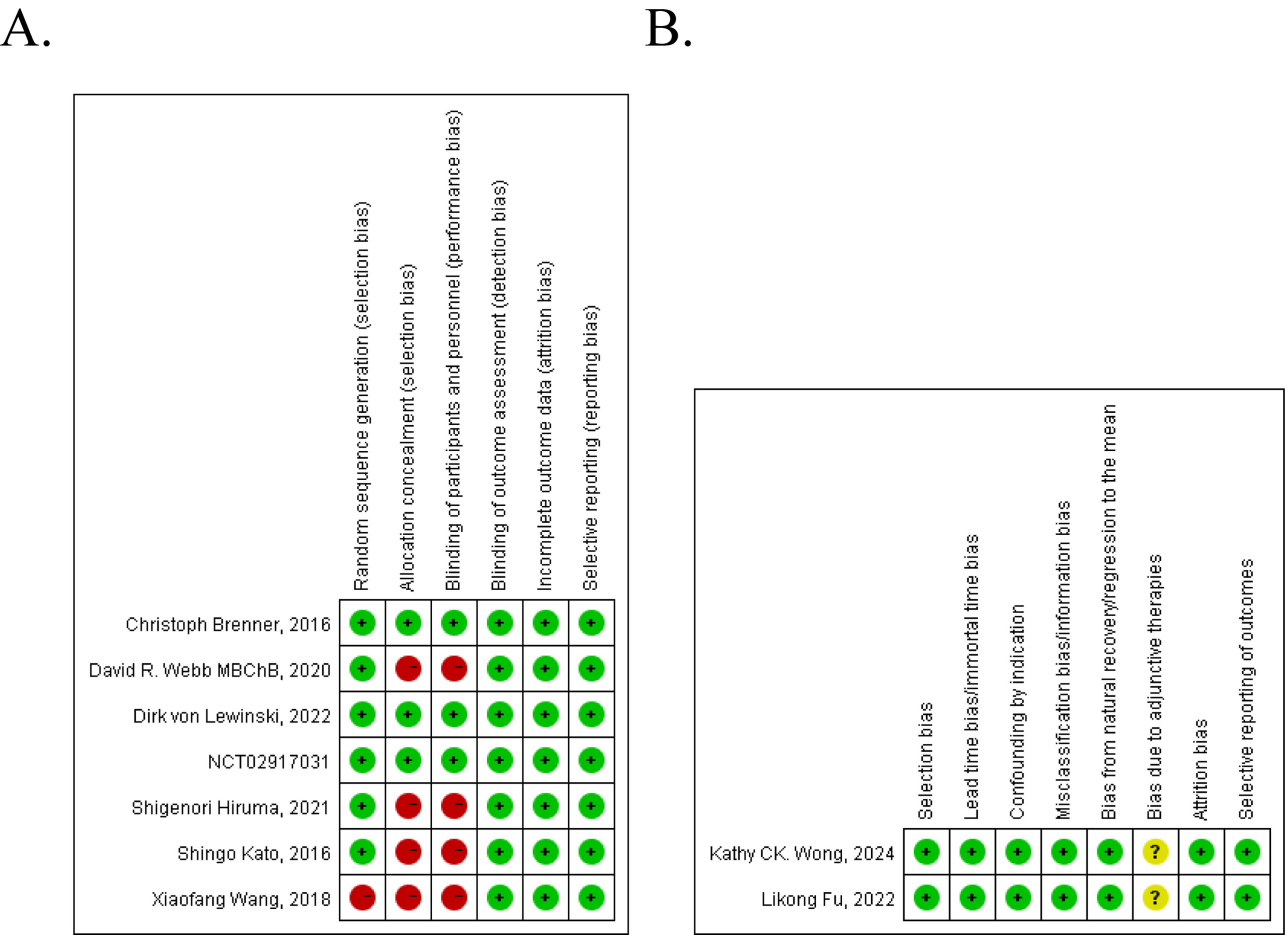
Risk of bias summary [**(A)** clinical control studies, **(B)** single-arm studies].

The quality assessment of these nine studies is shown in **Figure 2**. Of the seven controlled clinical studies, four studies got a point of 4 or less and were considered to be of poor methodological quality. The rest three studies were considered as good (**Figure 2A**). For two single-arm studies, 7 points were obtained, which we can consider to be of high methodological quality (**Figure 2B**).

### 3.3 Effect on global cardiac function

**Figure 3:**
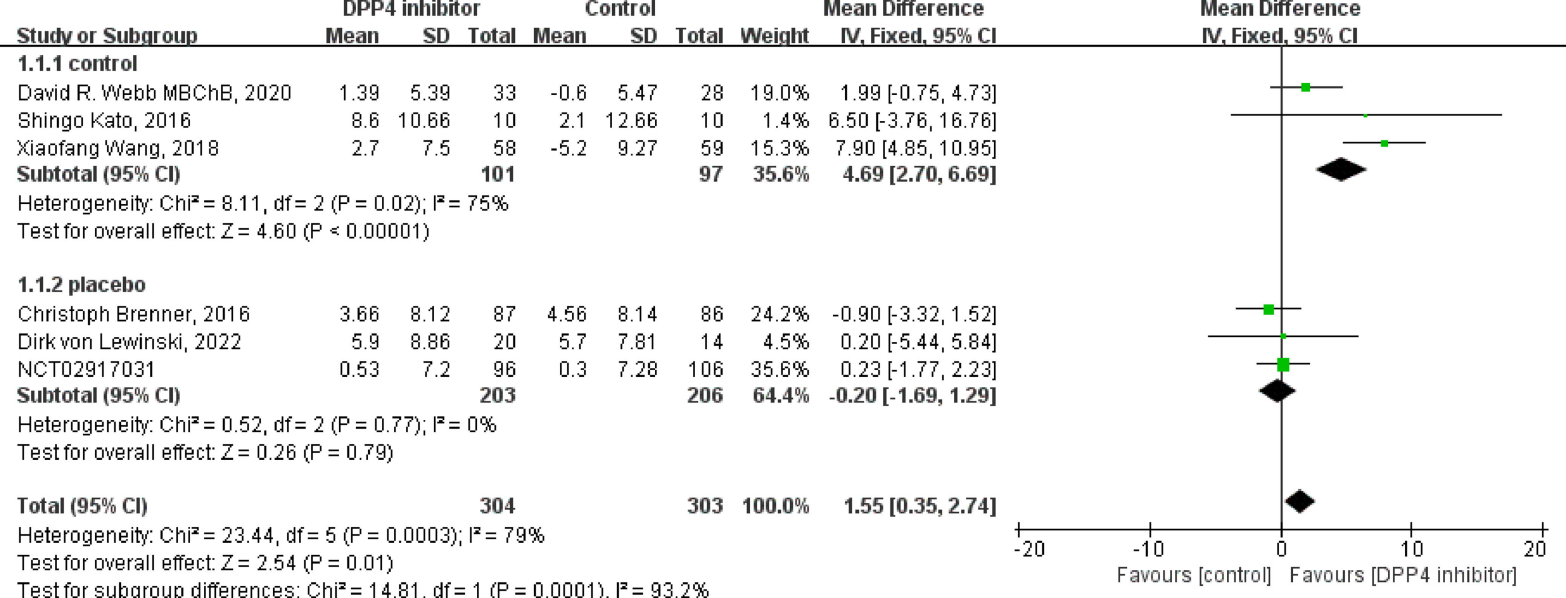
Forest plot of the LVEF through clinical control studies.

**Figure 4:**
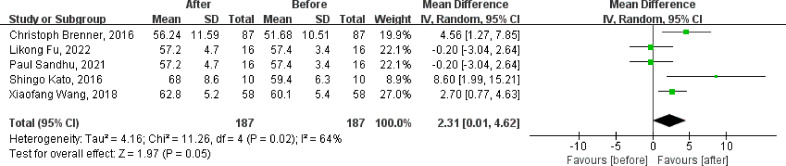
Forest plot of the LVEF through single-arm studies (self before-after comparison).

Of the nine included studies, eight studies reported the LVEF as an outcome, and six were clinical control studies (32, 35, 36, 38, 40, 41), five studies reported LVEF both before and after DPP4i treatment, respectively (32, 33, 37, 38, 41). Totally, for clinical control studies, the merged MD values of ΔLVEF by fixed-effect model and the pooled effect size in favor of DPP4i was 1.55 (95% CI 0.35 to 2.74, P=0.01). Heterogeneity analysis showed a huge heterogeneity (Chi^2^ = 23.44, P=0.0003, I^2^ = 79%) (**Figure 3**). Subgroup analysis was performed according to whether the control group was a placebo or not. Compared with positive control drugs, DPP4i can significantly improve the LVEF (MD=4.69, 95%CI=2.70 to 6.69), whereas, a big heterogeneity existed (Chi^2^ = 8.11, P<0.00001, I2 = 75%). However, there was no such change compared to placebo (MD=-0.20, 95%CI=-1.69 to 1.29). For single-arm studies and partial clinical control studies that reported LVEF values before and after DPP4i treatment, random-effect model was used to combine effect size due to a large heterogeneity (Chi^2^ = 11.26, P=0.02, I^2^ = 64%), and the pooled effect size in favor of DPP4i was 2.31 (95% CI 0.01 to 4.62, P=0.05), which indicated that DPP4i could improve LVEF (**Figure 4**). Overall, from the above results, we can still see the trend of DPP4i’s effect on improving LVEF.

Four studies reported the RVEF value (33, 36, 37, 41), two were clinical control studies (36, 41) and three analyzed the outcome both before and after DPP4i treatment (33, 37, 41). For clinical control studies, the merged MD values of △RVEF by fixed-effect model and the pooled effect size was 0.99 (95% CI -1.64 to 1.83, P=0.92). No heterogeneity was found in the heterogeneity test (Chi^2^ = 0.76, P=0.38, I^2^ = 0%). For three single-arm studies, no changes in RVEF were found before and after treatment (MD=-0.06, 95% CI -1.67 to 1.55, P=0.94), and no heterogeneity existed (Chi^2^ = 0.03, P=0.98, I^2^ = 0%). In total, DPP4i has no effect on RVEF.

### 3.4 Impact on further left ventricular structure and function parameters

Other outcome representing left ventricular structure and function parameters, such as left ventricular end-diastolic volume (LVEDV), left ventricular end-diastolic volume indexed (LVEDVI), left ventricular end-systolic volume (LVESV), left ventricular end-systolic volume indexed (LVESVI), left ventricular mass (LVM), left ventricular mass indexed (LVMI), peak filling rate (PFR) and time to peak filling rate (TPFR) have also been reported and been further analyzed here. Both in two types of studies, we have not observed the effect of DPP4i on the LVEDV, LVEDVI, LVESVI, LVESV, LVM and LVMI, namely DPP4i don’t change these left ventricular structure and function parameters (**Table 2**). It was found that DPP4i significantly increased the PFR without heterogeneity when the effect sizes of two single-arm studies were combined (MD=31.98, 95% CI 13.69 to 50.27, P=0.0006; heterogeneity test: Chi^2^ = 0.56, P=0.46, I^2^ = 0%) (32, 34). But this result has not been confirmed when combined the results from two clinical control studies (P=0.76) (32, 35).

**Table 2:** Summary of left ventricular structure and function parameters by CMR.

Index	Reference	Model	Mean difference	95%CI	*P* valve	*I* ^2^(%)
Clinical control studies						
LVEDV	1, 5	Fixed-effect	1.00	(-9.92,11.92)	0.86	0
LVEDVI	4, 5, 7, 9,10	Fixed-effect	-1.88	(-4.19,0.44)	0.11	0
LVESV	1, 5	Fixed-effect	0.51	(-6.52,7.54)	0.89	0
LVESVI	5, 7, 9,10	Fixed-effect	-1.15	(-3.46,1.17)	0.33	0
LVM	4, 5, 9	Fixed-effect	-2.38	(-5.87,1.11)	0.18	0
LVMI	4, 5, 7	Fixed-effect	-1.28	(-3.86,1.30)	0.33	0
PFR	1,4	Random-effect	12.70	(-68.08,93.48)	0.76	92
Single-arm studies						
LVEDV	1,2,6	Fixed-effect	3.65	(-3.90,11.20)	0.34	0
LVEDVI	2,6,7,10	Fixed-effect	0.06	(-3.12,3.24)	0.97	0
LVESV	1,2,6	Fixed-effect	-0.16	(-3.82,3.50)	0.93	0
LVESVI	2,6,7,10	Fixed-effect	-0.83	(-2.71,1.06)	0.39	0
LVM	2, 6	Fixed-effect	3.00	(-7.03,13.03)	0.56	0
LVMI	2,6,7	Fixed-effect	0.78	(-2.80,4.36)	0.67	0
PFR	1,3	Fixed-effect	31.98	(13.69,50.27)	0.0006	0
TPFR	1,3	Random-effect	Std. -0.45	(-1.17,0.27)	0.22	71

LVEDV, left ventricular end-diastolic volume; LVEDVI, left ventricular end-diastolic volume indexed; LVESV, left ventricular end-systolic volume; LVESVI, left ventricular end-systolic volume indexed; LVM, left ventricular mass; LVMI, left ventricular mass indexed; PFR, peak filling rate; TPFR, time to peak filling rate.

### 3.5 Impact on further right ventricular structure and function parameters

Only one clinical control study reported the change of right ventricular end-diastolic volume (RVEDV), right ventricular end-diastolic volume indexed (RVEDVI), right ventricular end-systolic volume (RVESV), right ventricular end-systolic volume indexed (RVESVI) compare to control group (36). At the same time, two single-arm studies reported the above indicators both before and after treatment (33, 37). Both two study types, DPP4i did not significantly change the above right ventricular structure and functional parameters and DPP4i also did not alter the RVM and RVMI in self-controlled single-arm trials. The details were shown in **Table 3**.

**Table 3:** Summary of right ventricular structure and function parameters by CMR.

Index	Reference	Model	Mean difference	95%CI	*P* valve	*I* ^2^(%)
Clinical control studies						
RVEDV	5	–	6.50	(-12.20,25.20)	0.50	–
RVEDVI	5	–	2.10	(-7.40,11.60)	0.66	–
RVESV	5	–	-0.30	(-13.88,13.28)	0.97	–
RVESVI	5	–	3.30	(-1.49,8.09)	0.18	–
Single-arm studies						
RVEDV	2,6	Fixed-effect	2.00	(-11.16,15.16)	0.77	0
RVEDVI	2,6	Fixed-effect	1.00	(-4.22,6.22)	0.71	0
RVESV	2,6	Fixed-effect	1.00	(-5.58,7.58)	0.77	0
RVESVI	2,6	Fixed-effect	0.00	(-2.71,2.71)	1.00	0
RVM	2, 6	Fixed-effect	0.00	(-2.26,2.26)	1.00	0
RVMI	2, 6	Fixed-effect	0.00	(-0.88,0.88)	1.00	0

RVEDV, right ventricular end-diastolic volume; RVEDVI, right ventricular end-diastolic volume indexed; RVESV, right ventricular end-systolic volume; RVESVI, right ventricular end-systolic volume indexed; RVM, right ventricular mass; RVMI, right ventricular mass indexed.

### 3.6 Other parameters

Shigenori Hiruma et al. reported the changes in accumulation of pericardial fat and myocardial triglyceride content between sitagliptin and empagliflozin groups (39). DPP4i did not significantly change the heart fat content when the statistics were pooled (accumulation of pericardial fat: MD=-79.8, 95%CI -190.13 to 30.53, P=0.16; myocardial triglyceride content: MD=0.80, 95%CI -2.49 to 4.09, P=0.63). Results of myocardial strain parameters (including global radial strain (GRS), global circumferential strain (GCS), and global longitudinal strain (GLS)) were reported in two single-arm studies (33, 37), but no changes were found.

### 3.7 Publication bias

**Figure 5:**
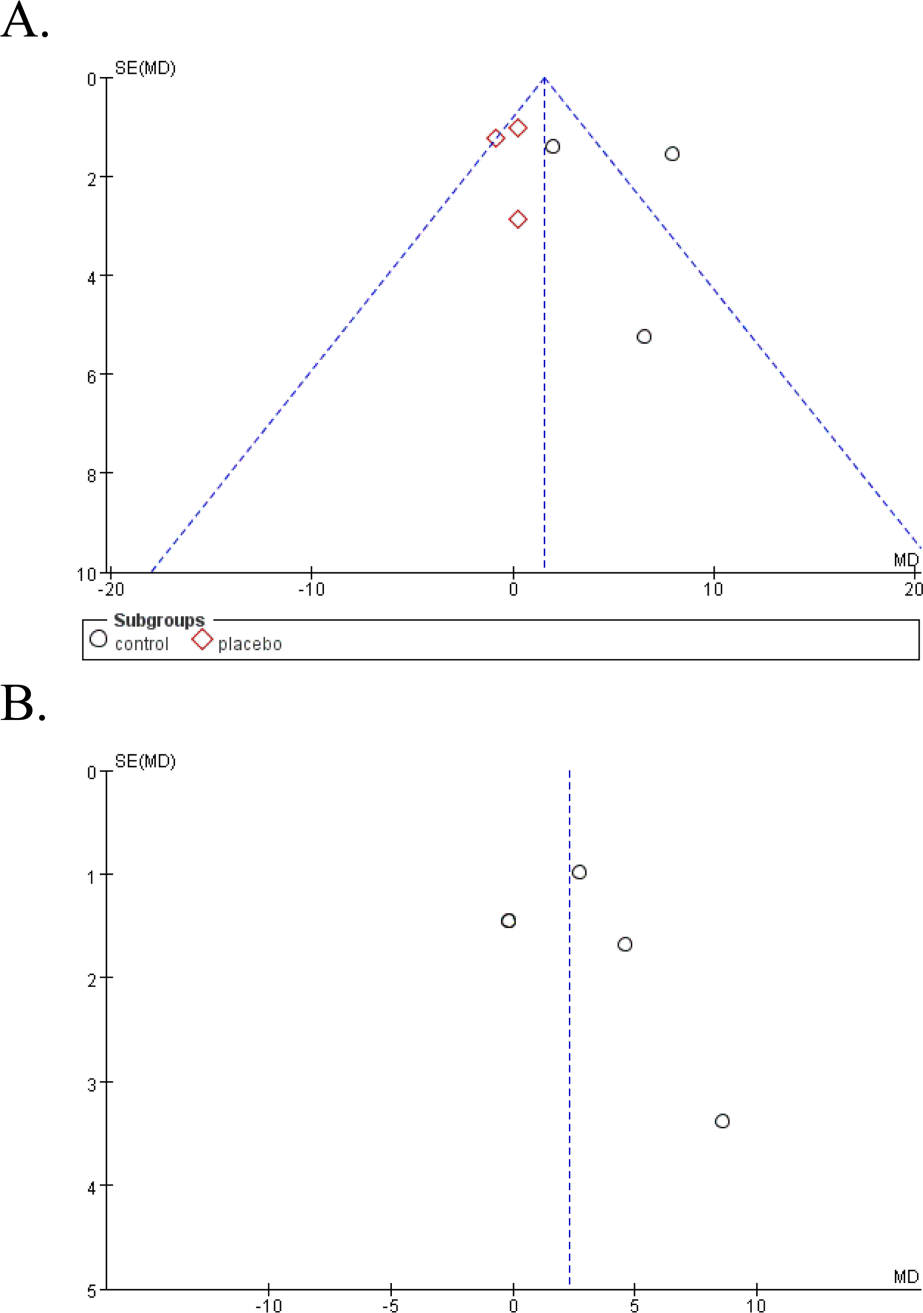
Funnel plot of publication bias [**(A)** clinical control studies, **(B)** single-arm studies].

Funnel plot was done to show the publication bias and results were shown in **Figure 5**. Due to the limited numbers of included studies, selection bias is significant but inevitable.

## 4 Discussion

Previous clinical studies have suggested the cardiac safety and even cardiac benefits of a variety of hypoglycemic drugs, including DPP4i. Through this study, we found that DPP4i can indeed improve the level of LVEF, and the results from single-arm studies suggest the potential role of DPP4i in improving PFR.

LVEF is an indicator of left ventricular systolic dysfunction, which is of great significance for the prognosis of heart disease. Studies conducted by Read et al. have shown that DPP4i can increase the LVEF assessed by dobutamine stress in patients with ischemic heart disease (42). In addition, echocardiography is a clinical first-line imaging examination of cardiovascular diseases, mainly used to evaluate the heart structure, function and hemodynamics, is currently the highest time resolution of non-invasive imaging technology, little affected by heart rate and rhythm. Compared with other imaging methods, echocardiography has the advantages of real-time, dynamic, convenient and economical, but low spatial resolution, low signal-to-noise ratio, small scanning field of view and large operator dependence are its main shortcomings. Obviously, the use of echocardiography to evaluate the effect of DPP4i on LVEF has also been adopted by some scholars, and the effect of DPP4i on improving LVEF has also been reported (32, 43). However, some studies have come to the opposite conclusion (44). Could this be due to the limitations of echocardiography? Compared with echocardiography, CMR has higher spatial resolution, can accurately delineate the endocardium and the epicardium, and then obtain more accurate cardiac function parameters, and it has become the recognized gold standard for non-invasive evaluation of cardiac structure and function (45, 46). Therefore, CMR was used to evaluate left ventricular systolic function in this study, which has a high sensitivity and makes the study results more accurate. However, the mechanism by which DPP4i increase the level of LVEF is not fully understand. According to Frank-Starling’s law, an increase in LVEDV leads to an increase in ejection fraction. However, our study found that despite an increase in LVEF, there was no increase in LVEDV. It has also been suggested that anti-inflammatory effect may be one of the mechanisms, but it has not been widely verified and recognized (38, 47). Another more accepted theory may be that because DPP4i can increase endogenous GLP-1 and glucose-dependent insulin stimulating polypeptide concentrations by inhibiting DPP4 enzyme activity, and enhance their effects. GLP-1 agonists have been shown to increase the level of LVEF (44). This may due to its role in regulating PI3K/Akt1 and AMPKα signaling that to inhibit angiotensin II and pressure overloading inducing cardiac remodeling (48). Wang et al. (49) also found that the cardioprotective effect of GLP-1 agonists may depend on the inhibition of oxidative stress through the mammalian target rapamycin complex 1/p70 ribosomal protein S6 kinase pathway. In experimental animal models of heart failure, GLP-1 agonists protect the heart during acute ischemia and improve mitochondrial function, microvascular function and myocardial glucose uptake (50, 51). GLP-1 agonists were found in left ventricular cardiomyocytes, thus it may have a direct effect on the ventricle (52). In addition, GLP-1 agonists can reduce inflammation, reduce ischemic injury, increase heart rate, promote plaque stabilization, and reduce smooth muscle proliferation (53). Studies have also shown that the positive effects of GLP-1 agonists on cardiovascular diseases may be the result of direct action on the arteriosclerosis process (54). Therefore, the principal mechanisms underlying this cardioprotection likely involve the suppression of cardiac oxidative stress, apoptosis, ferroptosis, necroptosis, and pyroptosis, which may also directly contribute to enhanced cardiac resistance to ischemia/reperfusion (I/R) injury (55). Furthermore, DPP4i mitigate the progression of diabetic microvascular and cardiovascular complications by reversing alterations associated with hyperglycemic memory. This process is linked to epigenetic modifications, which represent a significant area of current research within the context of metabolic memory phenomena related to diabetic complications (56).

PFR is an important indicator of diastolic function, which decreases when this function is impaired. Diabetic cardiomyopathy is strongly linked to diastolic dysfunction (57). Our research indicates that DPP4i can enhance PFR, highlighting its potential as a treatment for diastolic dysfunction. Earlier studies found that sitagliptin delayed left ventricular diastolic dysfunction in diabetic mice (58). In essence, DPP4i effectively enhances cardiac function.

However, this meta-analysis has some limitations. Only nine studies were included, potentially introducing biases and methodological errors due to their varying designs and poor methodological quality. The small number of studies in subgroup analyses could impact the findings’ reliability. Additionally, DPP4i directly affects ventricular function, but changes in body weight, blood pressure, and waist size could indirectly influence cardiometabolic parameters. While DPP4i’s cardiac benefits seem consistent in patients with or without CVD or HF history, but more research is needed to assess their advantages based on different HF and CVD risk levels due to limited clinical data.

## 5 Conclusions

In conclusion, the results of our meta-analysis show that DPP4i ameliorates PFR and LVEF levels in patients, as measured by CMR technology.

## Data Availability

The original contributions presented in the study are included in the article/supplementary material. Further inquiries can be directed to the corresponding author.
